# Sound Localization and Speech Enhancement Algorithm Based on Dual-Microphone

**DOI:** 10.3390/s22030715

**Published:** 2022-01-18

**Authors:** Tao Tao, Hong Zheng, Jianfeng Yang, Zhongyuan Guo, Yiyang Zhang, Jiahui Ao, Yuao Chen, Weiting Lin, Xiao Tan

**Affiliations:** School of Electronic Information, Wuhan University, Wuhan 430072, China; tt1295@whu.edu.cn (T.T.); yjf@whu.edu.cn (J.Y.); guozhongyuan@whu.edu.cn (Z.G.); monster233@whu.edu.cn (Y.Z.); jiahui@whu.edu.cn (J.A.); chenyuao@whu.edu.cn (Y.C.); 2019262120001@whu.edu.cn (W.L.); haiantanxiao@whu.edu.cn (X.T.)

**Keywords:** dual-microphone array, sound localization, speech enhancement, time delay estimation, post-filtering

## Abstract

In order to simplify the complexity and reduce the cost of the microphone array, this paper proposes a dual-microphone based sound localization and speech enhancement algorithm. Based on the time delay estimation of the signal received by the dual microphones, this paper combines energy difference estimation and controllable beam response power to realize the 3D coordinate calculation of the acoustic source and dual-microphone sound localization. Based on the azimuth angle of the acoustic source and the analysis of the independent quantity of the speech signal, the separation of the speaker signal of the acoustic source is realized. On this basis, post-wiener filtering is used to amplify and suppress the voice signal of the speaker, which can help to achieve speech enhancement. Experimental results show that the dual-microphone sound localization algorithm proposed in this paper can accurately identify the sound location, and the speech enhancement algorithm is more robust and adaptable than the original algorithm.

## 1. Introduction

Microphone array is a key technology of human–computer interaction (HCI). It can enhance the efficiency of HCI and adapt intelligent speech device to more complex and changing environments [1,2,3]. Microphone array, which can acquire the voice signal by microphones, uses digital electronic technology to sample, process and analyze the acoustic field characteristics, so that the collected voice signal is easier to be processed. Due to factors such as cost control, performance optimization, and environmental adaptability, acoustic signal processing based on dual-microphones is a challenging task [4,5].

Acoustic signal processing technology based on microphone array includes multiple technologies such as sound localization, speech separation, and speech enhancement. As a simple acoustic signal receiving device, the microphone is widely used in various sound localization experiments [6,7]. Ganguly A. et al. [8] proposed a dictionary-based singular value decomposition algorithm to solve the sound localization problem with the help of the non-linear and non-uniform microphone array in the smart phone and proved the accuracy of the algorithm in an environment with extremely low signal-noise ratio (SNR) through experimental results. Nevertheless, this algorithm cannot obtain the spatial position of the acoustic source and the distance from the acoustic source to the center of the microphone array. Jiaze Li and Jie Liu [9] derived and compared the four-element cross microphone array and the five-element cross microphone array based on the generalized cross-correlation time delay estimation algorithm. The experiment showed that the four-element cross microphone array has a blind spot for sound localization, while the five-element microphone can better improve localization accuracy and reduce errors. However, the structure of the microphone array is very complicated, which increases the hardware cost. Jelmer Tiete, Federico Domínguez, etc., [10] exploited the sensing capabilities of the Sound-Compass in a wireless sensor network to localize noise pollution, whose live tests produced a sound localization accuracy of a few centimeters in a 25 m^2^ anechoic chamber, while simulation results accurately located up to five broadband acoustic sources in a 10,000 m^2^ open field. The system requires 25 sensors, which makes it difficult to meet the requirements of miniaturization. Hongyan Xing, Xu Yang [11] made a theoretical model of a three-plane five-element microphone array is established, using time-delay values to judge the acoustic source’s quadrant position, which derived a formula for the sound azimuth calculation of a single-plane five-element microphone array based on sound geometric localization. It is also necessary to detect the environmental adaptability of the system and the working accuracy in a high-noise environment.

As the main method of acoustic signal processing, speech enhancement is a key issue to improve the accuracy of acoustic information extraction. Shujau M. et al. [12] proposed a multi-channel speech enhancement algorithm based on independent component analysis (ICA) for co-located microphone recording. Experiments show that the algorithm significantly improves the quality and clarity of the acoustic signal. This algorithm is more suitable for linear directional microphone arrays. Xunyu Zhu [13] advanced a deep neural network combining beamforming and deep complex U-net network to denoise acoustic signals from small-scale microphone arrays, which has certain advantages in environments such as homes, conference rooms, and classrooms. The author did not solve the human voice interference, especially the human voice interference that is in the same direction as the target acoustic source. On the basis of dual microphone arrays, Hairong Jia et al. [14] proposed a speech enhancement algorithm based on dual-channel neural network time-frequency masking, which combines single-channel neural network, adaptive mask orientation and proper positioning, and convolutional beamforming. Compared with traditional single-channel and dual-channel algorithms, the algorithm can extract voice information more clearly. In the algorithm, the network model has a large amount of calculation, which leads to higher hardware performance requirements for system implementation.

Traditional microphone arrays require many microphones, resulting in high cost and high design requirements. At present, there are new design concepts for intelligent home speech modules, such as lightweight, high integration and cost control. Speech module design based on dual microphones or even single microphone is an increasingly popular direction in the field of intelligent acoustic signal processing [15,16].

With the current lightweight and highly integrated design concepts of intelligent homes and interactive robots, combined with the current status and development trend of voice signal processing, this paper proposes a dual-microphone-oriented sound localization and voice enhancement optimization algorithm. The algorithm can use two microphones to locate the speaker target, realize the enhancement of the acoustic signal, and output a high SNR corpus that is more convenient for back-end analysis.

The rest of the paper is arranged as follows. Section 2 introduces two acoustic signal models in detail. Section 3 describes the improved algorithm of sound localization based on dual-microphone. Section 4 presents the advanced speech enhancement algorithm based on sound localization. Section 5 gives the experimental results. Section 6 concludes the paper.

## 2. Acoustic Signal Model

On the one hand, analyzed from the propagation mode, the acoustic signal is a longitudinal wave. That is to say, it is a wave in which the particles in the medium move along the direction of propagation. On the other hand, the acoustic signal can also be seen as a spherical wave. After vibration occurs at the acoustic source to generate an acoustic signal, the medium near the acoustic source appears accompanied by vibration, and the voice signal spreads around along with the medium simultaneously [17].

According to the distance between the acoustic source and the microphone array, the acoustic field model can be divided into two types: Near-field model and far-field model. The near-field model regards the voice signal as a spherical wave, and it considers the amplitude difference of the voice signal received by the sensors on the microphone array.

Generally, the near-field model and the far-field model are defined according to the relationship between the distance between the acoustic source and the center point of the microphone array element and the acoustic wavelength [18]:

(1) $$\{\begin{array}{c} L>\frac{{2d}^{2}}{\lambda_{\min}},\;\mathrm{the}\;\mathrm{far}-\mathrm{field}\;\mathrm{model}\\ L<\frac{{2d}^{2}}{\lambda_{\min}},\;\mathrm{the}\;\mathrm{near}-\mathrm{field}\;\mathrm{model} \end{array}$$

In Equation (1), L is the distance between the acoustic source and the center of the microphone array, and d is the aperture of the array element, and $\lambda_{\min}$ is the minimum wavelength of the current voice.

## 3. Sound Localization Algorithm by Dual-Microphone

### 3.1. Time-Delay Estimation

We can calculate the azimuth angle of the acoustic source by processing multi-channel signals based on sound localization algorithms. When calculating the azimuth angle, the phase difference of the signals received by the microphones at different positions is used to estimate the position of the speaker. Generally, because the distances between the acoustic source and the two microphones are not same, the arrival time difference of the acoustic wave is reflected in the waveform diagram as the phase difference of the voice waveforms received by the two microphones. The distance difference between the speaker and the microphone array is equal to the product of the acoustic signal propagate speed in the air and the relative delay between the two microphones. As mentioned in Section 2, the acoustic signal can be seen as propagating outward in the form of waves.

**Figure 1:**
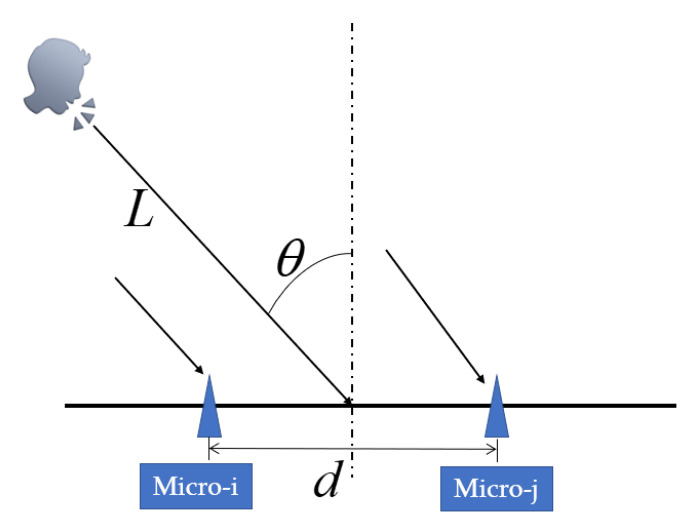
Principle of speaker positioning.

As shown in Figure 1, referring to the far-field model, we can estimate the azimuth in a 2D plane by dual-microphone array. However, if it is expanded to a 3D space, the estimated value of the azimuth angle will be a sector, so that the acoustic wave reaching the microphone is a spherical wave. At this time, the arrival angle θ cannot be expressed as a function of time delay, which is the difficulty of the sound localization algorithm based on dual microphones. After the time delay is obtained, the distance difference between the two microphones and the speaker can be calculated [19].

**Figure 2:**
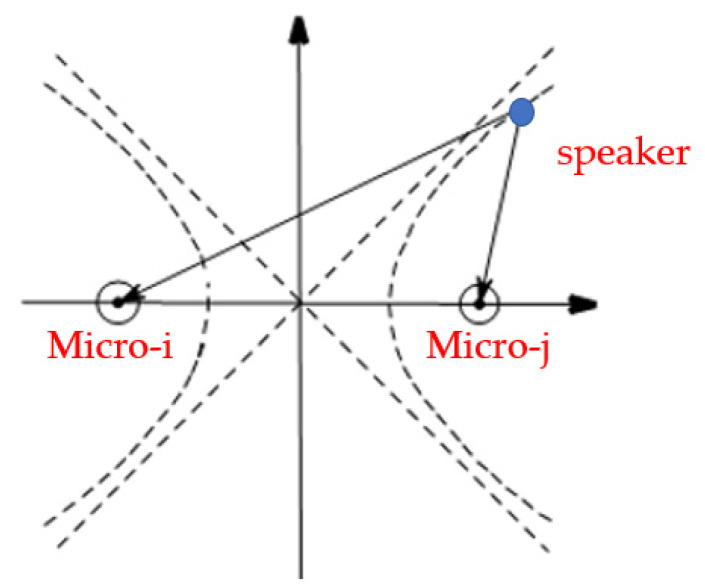
Dual-microphone positioning model.

**Figure 3:**
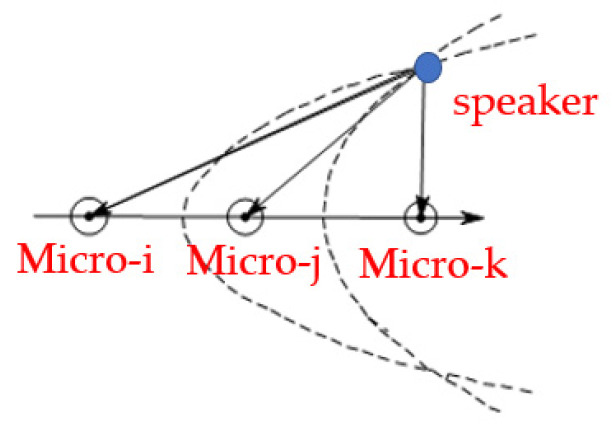
Multi-microphone positioning model.

As shown in Figure 2, the dual-microphone acoustic field is similar to the hyperbolic model. The distance difference between the point on the hyperbola and the two focal points is a fixed quantity, so the acoustic source must be located on the hyperbola. If there is another distance difference at the same time, the corresponding hyperbolas can also be calculated [20,21]. The intersection of the two hyperbolas is the speaker position, as shown in Figure 3.

Taking the midpoint of microphones i and j as the center of the coordinate system, the distance between microphones i and j is d. Let the coordinates of microphone i and j be (−d/2,0) and (d/2,0), respectively, and the acoustic source coordinates are (x,y). Then, the distance between the speaker and the two microphones is:

(2) $$\{\begin{array}{c} L_{\mathrm{is}}=\sqrt{{\left(\frac{d}{2}+x\right)}^{2}+y^{2}}\\ L_{\mathrm{js}}=\sqrt{{\left(\frac{d}{2}-x\right)}^{2}+y^{2}} \end{array}$$

In Equation (2), $L_{\mathrm{is}}$, $L_{\mathrm{js}}$ are the distance between the speaker and dual-microphone. The distance difference ${L\prime }_{\mathrm{ij}}=\left|L_{\mathrm{is}}-L_{\mathrm{js}}\right|$ between the speaker and the two microphones can be obtained. According to the estimated distance difference calculated by the time delay ${\hat{L\prime }}_{\mathrm{ij}}=c\times \Delta t_{\mathrm{ij}}$, the problem is transformed into the estimation of the position coordinate x of the speaker by minimizing the error between ${L\prime }_{\mathrm{ij}}$ and ${\hat{L\prime }}_{\mathrm{ij}}$ when the microphone coordinates and $\Delta t_{\mathrm{ij}}$ are known.

The sound localization method based on time delay requires at least two sets of data to construct two sets of hyperbolas and calculate their intersection points, because only the linear function relationship between x and y can be obtained by the information of time delay, which can only be embodied as the azimuth angle between the acoustic source and the center of the dual microphone array. If there is another distance difference exist, extra azimuth angle can be calculated by the new set of hyperbolas, and the intersection of the two hyperbolas is the acoustic source position. It can be seen that in a 2D space, three microphones can be used to estimate the position of the acoustic source. Therefore, the problem of sound localization is transformed to a problem of solving the intersection of two hyperbolas [22].

### 3.2. Energy Difference Estimation

In the process of acoustic wave propagation, there is energy attenuation except time delay exist. Considering the energy attenuation and time delay at the same time, the mathematical model of the signal received by the dual-microphones can be solved.

The acoustic signals received by microphones i and j are defined as follows:

(3) $$\{\begin{array}{c} x_{i}=\frac{2L(n-t_{i})}{d_{i}}\\ x_{j}=\frac{2L(n-t_{j})}{d_{j}} \end{array}$$

In Equation (3), x_i_ and x_j_ are the voice signals received by microphone i and microphone j, respectively. L(n) is the acoustic source, and t_i_ and t_j_ are the time when the two microphones receive signals. Respectively, d_i_ and d_j_ are the distances from the sound source to the two microphones. We define the sound intensity amplitude of the signal received by the microphone i as E_i_, which can be actually measured. Finally, the sound intensity amplitude is derived as shown in Equation (4).

(4) $$\frac{E_{i}}{E_{j}}=\frac{d_{i}^{2}}{d_{j}^{2}}$$

Combining Equations (3) and (4), we can get:

(5) $$\{\begin{array}{c} \sqrt{{\left(-\frac{d}{2}-x_{s}\right)}^{2}+{\left(0-y_{s}\right)}^{2}}=\frac{d_{ij}\sqrt{E_{j}}}{\sqrt{E_{j}}-\sqrt{E_{i}}}\\ \sqrt{{\left(\frac{d}{2}-x_{s}\right)}^{2}+{\left(0-y_{s}\right)}^{2}}=\frac{d_{ij}\sqrt{E_{i}}}{\sqrt{E_{j}}-\sqrt{E_{i}}} \end{array}$$

In Equation (5), d_ij_ is the distance difference between the sound source and the two microphones. It can be seen from Equation (5) that the two equations also construct a coordinate system similar to Figure 3 in the difference estimation of the energy field. In the energy field, the geometric model is two circles with the microphone i and j coordinates as the center and $d_{ij}\sqrt{E_{j}}/(\sqrt{E_{j}}-\sqrt{E_{i}})$, $d_{ij}\sqrt{E_{i}}/(\sqrt{E_{j}}-\sqrt{E_{i}})$ as the radius, respectively. The intersection of the two circles is the acoustic source position.

According to Euclidean geometry, when the distance between the centers of the two circles is greater than the difference between the radii of the two circles and less than the sum of the radii of the two circles, the two circles must intersect. Which is:

(6) $$d_{ij}=\frac{d_{ij}(\sqrt{E_{j}}-\sqrt{E_{i}})}{\sqrt{E_{j}}-\sqrt{E_{i}}}\leq d\leq \frac{d_{ij}(\sqrt{E_{j}}+\sqrt{E_{i}})}{\sqrt{E_{j}}-\sqrt{E_{i}}}$$

Obviously, Equation (6) is always established, so Equation (5) must have two sets of real number solutions that are symmetrical about the microphone connection. Finally, combined with the actual scene, the optimal solution in the 2D space is selected.

### 3.3. Sound Source Localization

Based on the time delay estimation and the energy difference estimation, the sound source position and the sound source direction angle under 2D coordinates will be obtained. However, it is still impossible to obtain the acoustic source distance in the 3D space.

In order to solve this problem, this paper introduces the Steered Response Power-Phase Transform (SRP-PHAT) based on the weighted phase transformation to achieve the maximum autocorrelation estimation, thereby obtaining the most likely acoustic source position in the 3D space.

Before the sound localization, pre-emphasis, framing and other pre-processing are performed on the acoustic signal. Based on short-time Fourier transform (STFT), the spectrum analysis of two single-channel speech signals is carried out with acoustic equal-frame modeling technique.

The PHAT algorithm in this paper uses a steerable beam response power algorithm to sum all possible phase transforms. SRP-PHAT can directly transform and process multi-channel microphone signals and use multiple microphones to improve the accuracy of position estimation.

SRP can be implemented using a block processing scheme that uses a short-time digital Fourier transform as an estimate of the microphone signal spectrum. Divide the array signal into blocks in the time domain and calculate the steering response for each block. The digital Fourier transform of the signal block is denoted by $X_{k,b}[k]$. Where, m is the microphone index, b is the block index, and $G_{k,b}[k]$ is the Fourier transform of the discrete-time filter of microphone m, which is performed separately in each block. The steering response of block b can be defined as follows:

(7) $${\tilde{P}}_{b}[\Delta_{1},\Delta_{2}]=\sum_{k=1}^{2}Y_{b^{\prime }}[k,\Delta_{1},\Delta_{2}]{\tilde{Y}}_{b}[k,\Delta_{1},\Delta_{2}]$$

${\tilde{Y}}_{b}[k,\Delta_{1},\Delta_{2}]$ is a discrete frequency function and successive steering delays with index k. Where, $\Delta_{1},\Delta_{2}$ represents all successive steering delays of the dual-microphone array in theory, it is necessary to process the data of all frequency bands in the signal. However, in actual, the data of one or more frequency bands are generally selected for processing. At the same time, although the k steering delays are continuous, in actual use, sampling is performed at a predefined set of spatial positions or directions, and the steering response power is obtained by summing k discrete frequencies.

(8) $${\tilde{Y}}_{b}[k,\Delta_{1},\Delta_{2}]=\sum_{k=1}^{2}G_{k,b}[k]X_{k,b}[k]e^{-jw\Delta_{k}}$$

The discrete filter G(t) is defined as Equation (9):

(9) $$G_{m,b}(k)=\frac{1}{F_{m,b}(k)},m=1,2$$

where, b is the block index after framing, $F_{m,b}(k)$ is the Fourier transform of the signal block after framing, m is the microphone index.

Substituting Equation (9) into Equation (7), the controllable response weighted by the phase transformation is expressed as:

(10) $${\tilde{Y}}_{b}^{PHAT}(\Delta_{1},\Delta_{2})=\sum_{k=1}^{2}\frac{F_{m,b}(k)}{\left|F_{m,b}(k)\right|}e^{-jw\Delta_{m}},m=1,2$$

Substituting Equation (10) into Equation (8), the controllable response power SRP-PHAT weighted by phase transformation can be obtained as:

(11) $${\tilde{P}}_{b}^{PHAT}(\Delta_{1},\Delta_{2})=\sum_{k=1}^{2}{\tilde{Y}}_{b}^{PHAT}(k,\Delta_{1},\Delta_{2}){\tilde{Y}}_{b^{\prime }}^{PHAT}(k,\Delta_{1},\Delta_{2})$$

In theory, it is necessary to analyze the data of all frequency bands in the acoustic signal. However, in the algorithm realization process, the acoustic signal processing method is somewhat different from the theory. Firstly, a predefined set of spatial positions or directions. Secondly, the voice signal is sampled, and the discrete frequencies are summed. Finally, the steering response power ${\tilde{P}}_{b}^{\mathrm{PHAT}}$ can be obtained.

**Figure 4:**
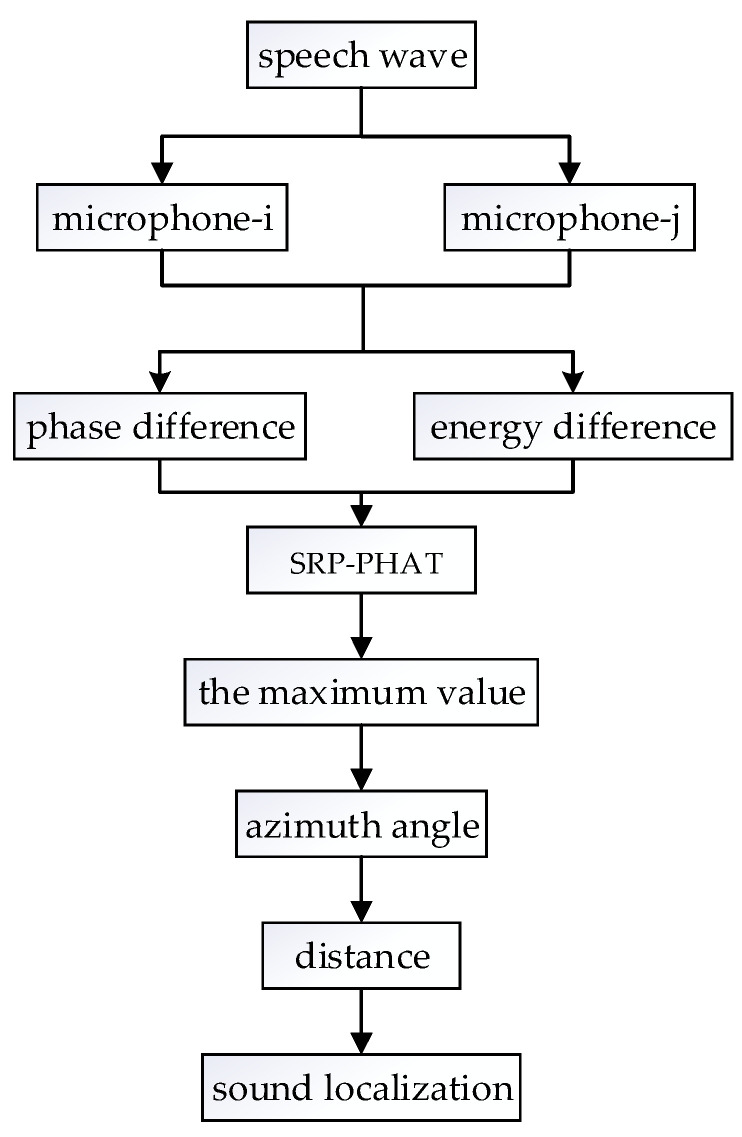
Sound localization algorithm based on dual-microphone.

The sound localization steps are as follows:

(1) Calculating the controllable time delay of the 2D azimuth direction in Section 3.2, which is according to the physical parameters of the microphone array;
(2) Using the STFT of the acoustic signal and the controllable time delay to calculate the SRP-PHAT for all frequencies in this direction;
(3) Repeating the above operations until SRP-PHAT in all directions is obtained;
(4) Selecting the direction corresponding to the maximum value as the azimuth angle of the sound source in 3D;
(5) Obtaining the 3D position of the sound source;
(6) The sound source localization algorithm flow is shown in Figure 4.

## 4. Speech Enhancement Algorithm Based on Sound Localization

Traditional algorithms have insufficient speech enhancement effects in strong-noise or multi-noise environments. Correlation noise will be generated and there are higher requirements for the microphone array. With the development of signal processing technology, more and more speech enhancement algorithms have emerged, such as wavelet transformation, speech enhancement algorithms based on empirical mode decomposition and deep learning [23]. New speech enhancement algorithms pay more attention to noise feature analysis and statistics. According to the analysis results of the noise characteristics, the noise signal and the original speech signal are separated to further obtain the original speech signal, but the algorithm time efficiency and economic efficiency are low.

Combining the results of sound localization in Section 3, this paper proposes an optimization algorithm for indoor speech enhancement based on post-filtering. According to the azimuth information of the acoustic source, the enhancement algorithm only amplifies the acoustic signal from the speaker, while other signals are judged as background noise and will be effectively suppressed.

### 4.1. Speech Separation Algorithm Based on the Azimuth of the Target Sound Source

The ultimate goal of speech enhancement technology is to extract the source signal, but the source signal is often unclear in the living environment, resulting in the speaker signal entraining other interference signals or noise during the enhancement process [24]. The speech separation algorithm can not only remove the environmental noise and interference components, but also effectively separates the speech signals of different speakers. Independent Component Analysis (ICA) has better performance and higher stability, which is currently the most conventional and popular speech separation algorithm [25,26]. The principle of ICA is decomposing the aliased signal to obtain several independent signals. In this article, we define multiple independent source signals as S and the observation signal X after passing through the mixing matrix A, which is expressed as a matrix:

(12) X(t)=AS(t)

where, the observation signal X(t) is the linear aliasing of n mutually independent unknown source signals S(t), and A is an m×n aliasing matrix whose aliasing weight coefficient of the matrix is unknown. When both S(t) and A are unknown, the core of the ICA algorithm is to solve the demixing matrix W so that the final output signal Y(t) optimally approximates the source signal S(t) according to certain criteria (such as independence criteria):

(13) Y(t)=WX(t)

The process of solving the demixing matrix W is the process of feature extraction. This paper selects the azimuth angle information between acoustic source and dual-microphone as ICA analysis feature. Based on the definition of negative entropy, need to define was a column vector of matrix W. The objective function of the ICA algorithm is:

(14) $$J(w)\propto {\left\{E[G(w^{T}X)]-E[G(u)]\right\}}^{2}$$

where, u is a Gaussian variable with zero mean unit variance; G is a random non-negative quadratic function; X is a target sound source position vector, which is as the signal characteristic value. Taking the partial derivative of Equation (14) to get:

(15) $$\frac{\partial J}{\partial w}=2\left\{E[G(w^{T}X)]-E[G(u)]\right\}E[Xg(w^{T}X)]$$

In Equation (15), the g function is the derivative of the G function. Setting ∂J/∂w=0 directly will lead to poor convergence of the algorithm. Associating Equation (14) and Equation (15), it shows that the maximum value of the objective function J(w) can be obtained by the optimal solution of $E[G(w^{T}X)]$.

According to KKT constrained optimization, the optimal solution of $E[G(w^{T}X)]$ is an unconstrained optimization problem:

(16) $$J^{\prime }(w)=E[G(W^{T}X)]+\psi \left(\left\|w\right\|-1\right)$$

where, ψ is a constant parameter. Based on Equation (16), the function H(w) is defined as follows:

(17) $$H(w)=E[Xg(w^{T}X)]-\psi w$$

Derivation:

(18) $$\frac{\partial H}{\partial w}=E[XX^{T}g^{\prime }(w^{T}X)]-\psi$$

Finally, the matrix W is solved according to the Newton iteration method:

(19) $$\{\begin{array}{c} w(j+1)=E\{Xg[w^{T}(j)X]\}-E\{g^{\prime }[w^{T}(j)X]\}w(j)\\ w(j+1)=\frac{w(j+1)}{{\left\|w(j+1)\right\|}_{2}} \end{array}$$

### 4.2. Speech Enhancement Algorithm Based on Post-Adaptive Filter

The idea of sub-frame block-index in Section 3.3 will also be applied to the adjustment of Wiener filter parameters in speech enhancement. The core of the adaptive algorithm is to modify the parameters of the filter based on the analysis of the first three voice framing blocks of the dual-channel voice signal collected by the front-end dual microphones, so as to achieve the optimal filtering.

Spectral subtraction is one of the effective technologies to enhance the quality of the voice signal, it has a good noise reduction effect at low SNR, the convergence rate and imbalance are affected by step size in LMS adaptive filtering algorithm. This paper introduces a method to enhance the quality of speech signal based on the combination of spectral subtraction and variable-step LMS adaptive filtering algorithm, to adjust the step size by changing the squared term of error, the step size follows the principle of change after the first fixed, achieves the purpose to improve the convergence rate and reduces the steady-state error.

**Figure 5:**
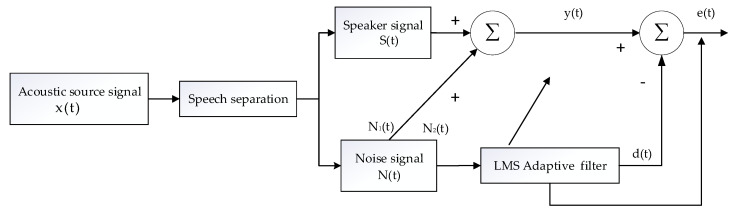
Adaptive filter flow.

As shown in Figure 5, x(t) is the original signal input, y(t) is the output signal of the system after the adaptive filter, e(t) is the expected response, and N(t) is the noise signal of the signal.

The key to adaptive noise filtering is to obtain the best estimate of noise. The filter parameters obtained from the previous speech frame are used to adjust the control parameters of the latter speech frame, so as to obtain the error function of the system for improving the SNR. If the reference noise is related to the noise in the signal, the randomness of the noise can be better offset, and the noise can be completely eliminated. However, when the reference noise is not correlated with the noise in the signal or the correlation is weak, the noise cannot be completely cancelled out, and the filtering effect is not obvious. From Figure 5, we can get:

(20) $$e(t)=y(t)-d(t)=S(t)+N_{1}(t)-d(t)$$

Then:

(21) $$e^{2}(t)=S^{2}(t)+{\left[N_{1}(t)-d(t)\right]}^{2}+2[N_{1}(t)-d(t)]S(t)$$

Equation (21) takes the expectation on both sides of the equal sign to get:

(22) $$E[e^{2}(t)]=E[s^{2}(t)]+E{\left[N_{1}(t)-d(t)\right]}^{2}+2E[(N_{1}(t)-d(t)\cdot S(t)]$$

Since S(t) is not related to $N_{1}(t)$, and S(t) is not related to $N_{2(}t)$, 2E[(N(t)-d(t)·S(t)]=0:

(23) $$E[e^{2}(t)]=E[s^{2}(t)]+E{\left[N_{1}(t)-d(t)\right]}^{2}$$

The weight coefficient is adjusted by the LMS adaptive filter to obtain the minimum point of the nonlinear function $E[e^{2}(t)]$. When the value of $E[e^{2}(t)]$ in Equation (23) is minimum, the value of $E{\left[N_{1}(t)-d(t)\right]}^{2}$ in Equation (23) is also minimum. When the value of E[s^2^(m)] does not change, the output of the adaptive filter d(t) is the best estimate of N_1_(t), and the system output is:

(24) e(m)=s(t)+N(t)-d(t)

In this way, when the value of d(t) is closest to the value of N(t), the output of the adaptive LMS filter is e(m) = s(m).

**Figure 6:**
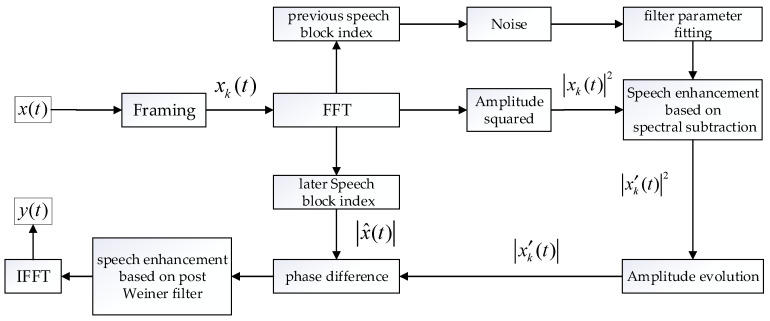
Adaptive speech enhancement flow.

This paper proposes a speech enhancement algorithm based on post Wiener filtering. First, the algorithm uses spectral subtraction to perform speech enhancement on the speech signal of the current sound source, which will obtain acoustic signal containing autocorrelation noise. Then, the parameters of the post-wiener filter are used to suppress noise and amplify the target of the sound source signal. Finally, the algorithm fits the optimal filter. The principal flow chart of the optimization algorithm is shown in Figure 6.

## 5. Experiment Design and Result Analysis

In order to verify the real performance and effectiveness of the algorithm in this paper, two experiments were designed. Experiment-I verifies the performance of this algorithm in real sound localization. Selecting some sound source points, we calculate the sound source position and compare it with the existing sound source localization method (based on the TDOA algorithm). Experiment-∏ is to verify the effectiveness of the proposed new method in speech enhancement. We process speech signals in different noise environments and compare them with other speech enhancement algorithms.

**Figure 7:**
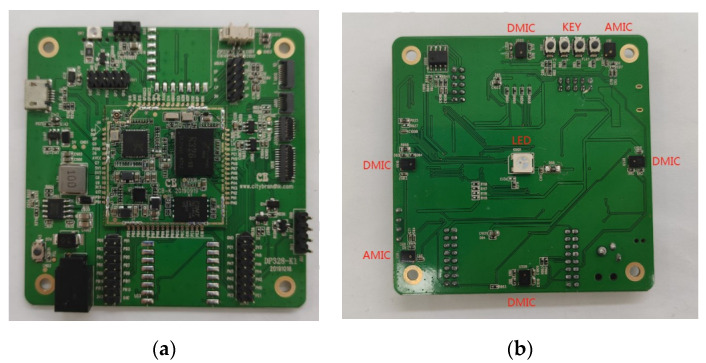
Allwinner R328 microphone array physical picture: (**a**) the front of Allwinner R328 microphone array; (**b**) the back of Allwinner R328 microphone array.

The experimental hardware uses the Allwinner R328 microphone array. Allwinner R328 relies on the computing ability of the cost-effective dual-core CortexTM-A7 CPU to provide the best computing ability at the lowest cost. The highly integrated CODEC can support key voice pick-and-place solutions without external DSP voice chip circuits. As shown in Figure 7, the Allwinner R328 microphone array has six microphones, including two digital microphones and four analog microphones. The back of Allwinner R328 microphone array also has four keys to adjust the recording volume and a LED to indicate that the device is working normally. In the experiment, only two digital microphones were used for recording. The distance between the two digital microphones is 15 cm, so the value of d in Equation (2) is 0.2.

When the voice signal is sampled, the two digital microphones on the array are used as recording devices. The distance between the two microphones is 20 cm, and the sampling rate is 16 KHz.

The experimental site was chosen as a hall of 10 m × 15 m × 4 m. The early reverberation time of the room is calculated to be 15 m through experiments. In living environment, there are many kinds of noises such as other people talking, air conditioners, and computer fans.

### 5.1. Acoustic Localization Experiment by Dual-Microphone

Firstly, we build a test prototype for the collection of the circular microphone array, and the programming the development board. In the experiment, the USB interface is used to connect with the PC, which not only supplies power to the hardware circuit, but also transmits the processed voice signal to the computer.

To test the dual-microphone sound source localization function, there are other speakers speaking in the laboratory to interfere with the target speaker’s voice signal, while one audio is also set to play different volume of interference noise (the noise level is divided into three levels according to the volume of the sound, and the three-level noise interference is the most serious).

Determining the accuracy of the acoustic azimuth angle measurement, the target speaker stands at different angle positions 4 m away from the center of the microphone. In the serial port tool, entering the relevant commands, the development board will record the target speaker and calculate its azimuth angle. In the actual positioning experiment, the measurement is repeated five times at each experimental point, and the average value is taken as the final positioning result of the point.

**Figure 8:**
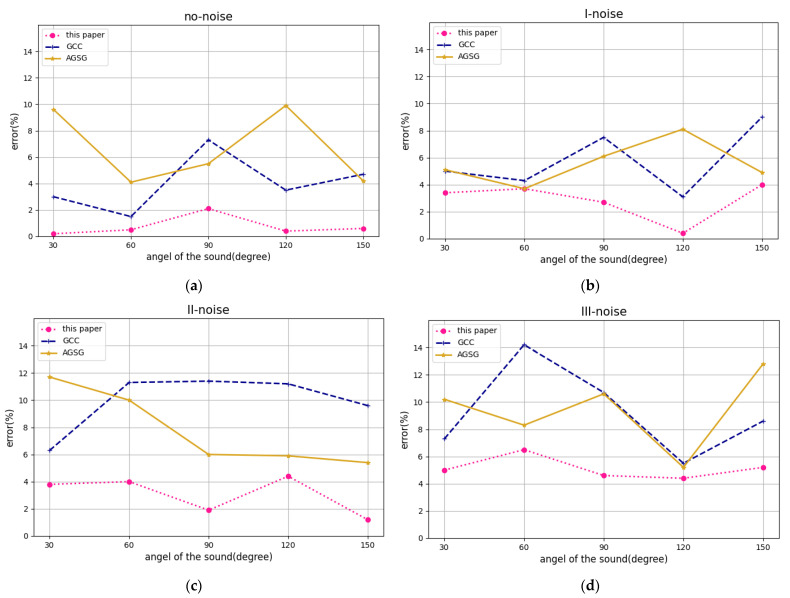

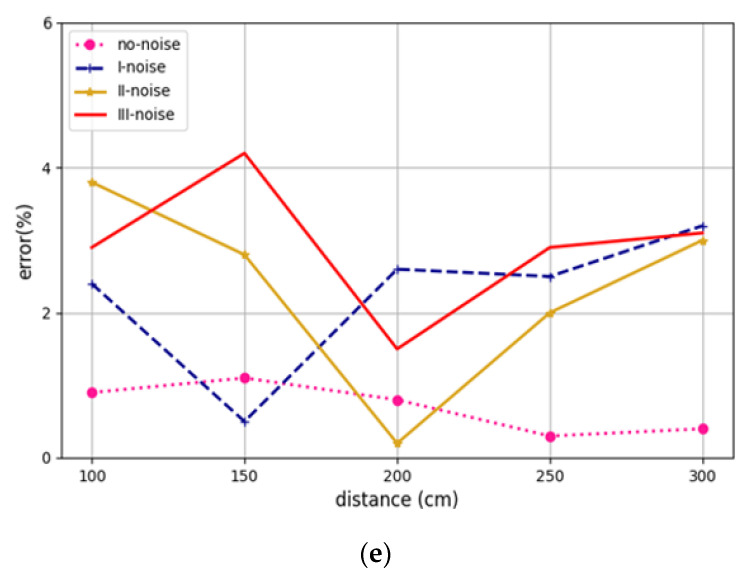
The experiment result of localization experiment: (**a**) the comparison of azimuth error around no-noise; (**b**) the comparison of azimuth error around I-noise; (**c**) the comparison of azimuth error around II-noise; (**d**) the comparison of azimuth error around III-noise; (**e**) distance error around noise for this paper.

We then conduct experiments on the accuracy of sound source distance measurement. Under four noise environments, the target speaker stands at the same angular position from different distances to the center of the microphone. Then we use the previous method to perform recording and 3D distance calculation. Similarly, the measurement is repeated five times at each experimental point, and the average value is taken as the final positioning result of the point. The experimental results are shown in the Figure 8.

### 5.2. Speech Enhancement Experiment

#### 5.2.1. Known Noise Simulation Experiment

In the simulation experiment, we will select 20 groups of speech files in a noise-free scene as clean speech signal. There are 4 kinds of noise in NOISE-92, which are babble, street, car and train. The SNR of added noise are −5 dB, 0 dB, and 5 dB. The sampling rate is 16 kHz. The quantization precision is 16 bits.

Perceptual evaluation of speech quality (PESQ) is an objective, full-reference voice quality assessment method. The PESQ algorithm requires a noisy attenuated signal and an original reference signal, which can provide an evaluation criterion for speech. The PESQ score is from −0.5 to 4.5. The higher the score, the better the voice quality.

Table 1 shows the quality value of noisy speech (not enhanced by the enhancement algorithm), the quality value enhanced by the GCC/AGSC algorithm, and the quality value after speech enhancement algorithm proposed in this paper. The processing standard of the two algorithms is controlled in the same way, and this quality value is the average of the 20 groups of speech files.

It can be seen from the table that the algorithm proposed in this paper has a higher quality value than the noisy speech and GCC/AGSC algorithm under all noise conditions, which proves that the algorithm proposed in this paper can greatly improve the enhanced speech quality. We compared the PESQ between the algorithm proposed in this paper and the GCC/AGSC algorithm to intuitively show the improvement of PESQ. From Table 1, it is clear that the PESQ are increased by the algorithm proposed in this paper is improved under all three kinds of SNR conditions. Except for babble, the lower the signal noise ratio, the higher the quality value. The algorithm proposed herein is more advantageous to improve the quality of the speech under low signal noise ratio.

#### 5.2.2. Unknown Noise Reality Simulation Experiment

In the speech enhancement experiment, the acoustic files in the first-level noise and the third-level noise environment are selected to perform subsequent enhancement processing on the acoustic signal. According to the foregoing, the azimuth angle information of the sound source is used as a feature vector for acoustic signal separation. The voice system will only amplify the voice signal from this position and suppress other signals to achieve voice enhancement. Finally, the advantages of the algorithm in this paper are demonstrated through comparative experiments.

**Figure 9:**
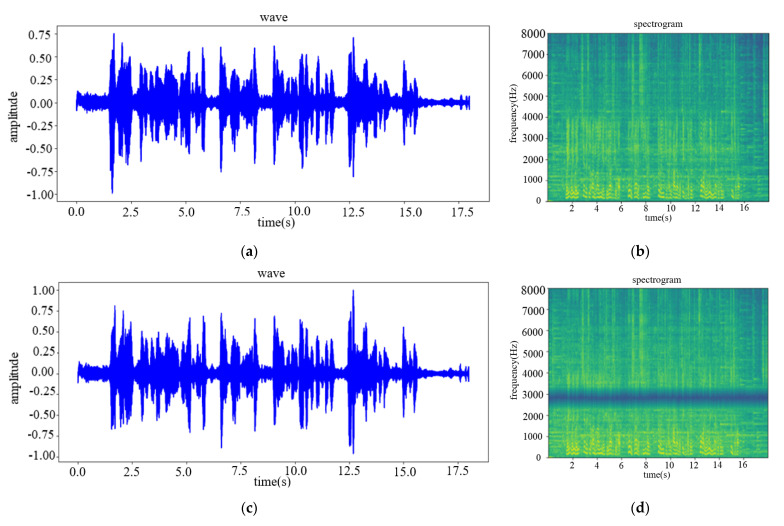

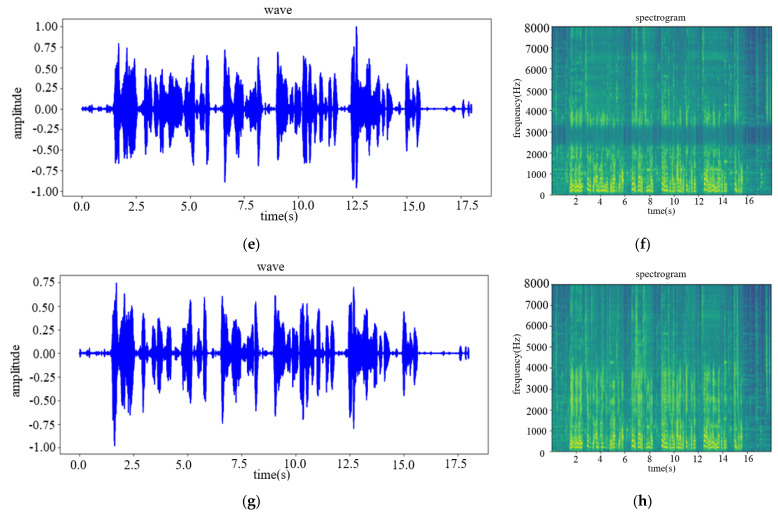
The results of speech enhancement comparison experiment in high-noise: (**a**) Original speech wav; (**b**) Original speech spectrogram; (**c**) GCC-enhanced speech wave; (**d**) GCC-enhanced speech spectrogram; (**e**) AGSC-enhanced speech wave; (**f**) AGSC-enhanced speech spectrogram; (**g**) the speech wave enhanced by the algorithm in this paper; (**h**) the speech spectrogram enhanced by the algorithm in this paper.

**Figure 10:**
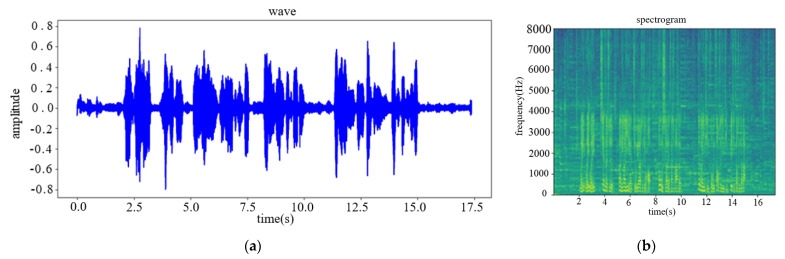

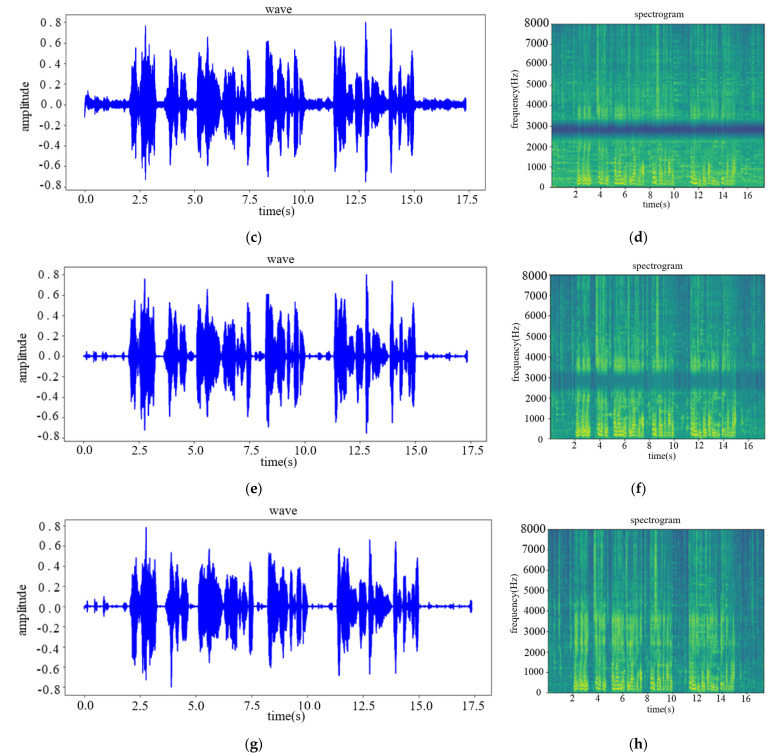
The results of speech enhancement comparison experiment in low-noise: (**a**) Original speech wave; (**b**) Original speech spectrogram; (**c**) GCC-enhanced speech wave; (**d**) GCC-enhanced speech spectrogram; (**e**) AGSC-enhanced speech wave; (**f**) AGSC-enhanced speech spectrogram; (**g**) the speech wave enhanced by the algorithm in this paper; (**h**) the speech spectrogram enhanced by the algorithm in this paper.

This paper uses the experimental data to test the technical solution in the laboratory and compares speech enhancement effect of the algorithm in this paper with GCC algorithm and AGSC algorithm. Figure 9 shows the high-noisy experimental speech and the output results of the two algorithms. Figure 10 shows the low-noisy experimental speech and the output results of the two algorithms.

From the comparison of the spectrogram, it can be found that when the GCC algorithm and the AGSC algorithm enhance the dual-channel speech signal, there will be auto-correlation noise and speech distortion; while the speech enhancement algorithm in this paper has neither obvious auto-correlation noise nor speech distortion. In addition, from the speech waveform information, the GCC algorithm and the AGSC algorithm have no accuracy of the sound source azimuth estimation with low SNR of the acoustic signal, which affects the speech enhancement performance. While the speech enhancement algorithm in this paper has better effect of background noise reduction and acoustic source target signal amplification.

Finally, in order to verify the comprehensibility of the corpus enhanced by the algorithm in this paper, eight speech files in the experiment were sequentially used for speech recognition by the speech transcribing module of iFLYTEK. In each speech file, the speaker said a total of 52 Chinese characters. The correct rate of speech recognition for each corpus is shown in Table 2.

## 6. Conclusions

This paper proposes an improved sound source localization and speech enhancement algorithm. By introducing the maximum controllable response power, based on the traditional time delay estimation, combined with the energy attenuation estimation, only two microphones are needed to complete the position settlement of the sound source in the three-dimensional space, which simplifies the design complexity and reduce cost of the microphone array. It also improves the accuracy of the sound source localization algorithm. Then the results of sound source localization are used to realize speech separation based on the azimuth of the target speaker, and complete speech enhancement based on adaptive filtering, and output a corpus with a higher SNR. Finally, related experiments are completed in combination with actual scenarios and hardware construction. The experimental results show that the dual-microphone-based sound source localization and speech enhancement algorithm proposed in this paper has extremely high accuracy and robustness. Compared with other speech enhancement algorithm, the corpus enhanced by the algorithm in this paper has a higher SNR.

## Figures and Tables

**Table 1:** The comparison of the PESQ value.

The Type of Noise	SNR	The Speech with Noise	GCC-Enhanced Speech	AGSC-Enhanced Speech	The Speech Enhanced by the Algorithm in This Paper
babble	−5 dB	1.38	1.35	1.53	1.74
	0	1.69	1.55	1.59	1.85
	5 dB	2.12	1.97	1.98	2.26
street	−5 dB	1.23	1.15	1.28	1.55
	0	1.72	1.61	1.80	1.96
	5 dB	2.21	2.16	2.27	2.29
car	−5 dB	1.48	1.33	1.46	1.77
	0	1.89	1.67	1.92	1.91
	5 dB	2.45	2.44	2.43	2.63
train	−5 dB	1.27	1.30	1.28	1.46
	0	1.56	1.67	1.66	1.91
	5 dB	2.17	2.18	2.15	2.52

**Table 2:** The correct rate of speech recognition.

Type	Test Environment	Correct Rate (%)
Original speech file	low-noise	67.31
	high-noise	57.69
GCC-enhanced speech file	low-noise	80.77
	high-noise	73.77
AGSC-enhanced speech file	low-noise	90.38
	high-noise	78.85
the speech file enhanced by the algorithm in this paper	low-noise	100
	high-noise	98.77
